# Identification of possible genetic alterations in the breast cancer cell line MCF-7 using high-density SNP genotyping microarray

**DOI:** 10.4103/1477-3163.50886

**Published:** 2009-05-06

**Authors:** Hui-Yun Wang, Danielle Greenawalt, Xiangfeng Cui, Irina V Tereshchenko, Minjie Luo, Qifeng Yang, Marco A Azaro, Guohong Hu, Yi Chu, James Y Li, Li Shen, Yong Lin, Lianjun Zhang, Honghua Li

**Affiliations:** 1 Department of Molecular Genetics, Microbiology and Immunology/The Cancer Institute of New Jersey, Piscataway, New Jersey, 08854, USA; 2 Department of Biometrics/The Cancer Institute of New Jersey, University of Medicine and Dentistry of New Jersey-Robert Wood Johnson Medical School, Piscataway, New Jersey, 08854, USA; 3 Department of Computer Science, University of Maryland, Baltimore County, Baltimore, Maryland 21250, USA; 4 Department of Forest and Natural Resources Management, College of Environment Science and Forestry, State University of New York, One Forestry Drive, Syracuse, New York, 13210, USA

**Keywords:** Genetic alteration, single nucleotide polymorphism, genotyping, MCF-7 cell line, loss of heterozygosity, microarray

## Abstract

**Context:**

Cancer cell lines are used extensively in various research. Knowledge of genetic alterations in these lines is important for understanding mechanisms underlying their biology. However, since paired normal tissues are usually unavailable for comparison, precisely determining genetic alterations in cancer cell lines is difficult. To address this issue, a highly efficient and reliable method is developed. Aims: Establishing a highly efficient and reliable experimental system for genetic profiling of cell lines.

**Materials and Methods:**

A widely used breast cancer cell line, MCF-7, was genetically profiled with 4,396 single nucleotide polymorphisms (SNPs) spanning 11 whole chromosomes and two other small regions using a newly developed high-throughput multiplex genotyping approach.

**Results:**

The fractions of homozygous SNPs in MCF-7 (13.3%) were significantly lower than those in the control cell line and in 24 normal human individuals (25.1% and 27.4%, respectively). Homozygous SNPs in MCF-7 were found in clusters. The sizes of these clusters were significantly larger than the expected based on random allelic combination. Fourteen such regions were found on chromosomes 1p, 1q, 2q, 6q, 13, 15q, 16q, 17q and 18p in MCF-7 and two in the small regions.

**Conclusions:**

These results are generally concordant with those obtained using different approaches but are better in defining their chromosomal positions. The used approach provides a reliable way to detecting possible genetic alterations in cancer cell lines without paired normal tissues.

## Introduction

Cancer cell lines have been used extensively in various cancer research. Knowledge of genetic aberrations in these cell lines is important for understanding mechanisms underlying biological behaviors of the cells including their responses to drugs. However, a large portion of genetic alterations such as those caused by loss of heterozygosity (LOH) and chromosomal segment amplification may not be precisely determined without comparing with the genotypes of paired normal tissue, which usually are unavailable.

MCF-7 is one of the widely used cancer cell lines. It was established 30 years ago[1] from a pleural effusion taken from a woman with metastatic breast carcinoma, and has been used worldwide for studying various aspects of breast cancer. Many studies on genomic variation of MCF-7 have been reported during the past 30 years. The methods employed in these studies include the conventional karyotyping,[2,3] metaphase[4,5] and interphase[6,7] in situ hybridizations (ISHs), comparative genomic hybridization [CGH],[8,9–11] and end-sequence profiling [ESP].[12] With these methods, a number of diverse genetic abnormalities in the MCF-7 cell line have been revealed, including chromosomal rearrangements, deletions, translocations, inversions, breakpoints and changes in chromosome and chromosomal segment copy numbers (loss or amplification). However, because all above methods, except ESP, are limited in resolution, the regions affected by the aberrations could not be precisely determined. Although ESP can reach high resolution, it involves complicated, laborious and large amounts of molecular cloning and sequencing, which is not suitable for the analysis of a large number of cell lines or tissue samples.

Extensive chromosomal aberrations in the MCF-7 genome were identified with CGH.[8–10,13–15] However, the conventional CGH requires preparation of chromosomes in metaphase spreads, and its resolution is limited to 10 ∼ 20 Mb for deletion and 2 Mb for amplification.[16,17] Recently, microarray-based CGH has emerged for high-resolution and high-throughput analysis.[18–20] Depending on the number and sizes of the clones used, microarray-based CGH could be used for genetic analysis at different resolutions. When cDNA clones are used, only the coding sequences can be included, not promoters, introns and intergenic sequences.[17] Since genomic clones contain all kinds of repeats with varying numbers, results from microarray based CGH may be complicated. This has been addressed at least in part recently by using sophisticatedly selected oligonucleotides as probes. However, since CGH is a DNA copy number based assay, it cannot be used to detect genetic aberrations caused by somatic recombination, one of the causes of LOH, which results in no copy number change in the affected regions. Furthermore, in many tumors, cells are very heterogeneous. In order to obtain accurate results, cell populations, which are often microscopic in size, must be isolated by microdissection and the amount of material available for analysis is usually very small. In this case, CGH cannot be used unless unbiased whole-genome amplification is used before CGH analysis.

The advent of high-throughput SNPs genotyping technologies has made it possible to use single nucleotide polymorphism (SNP) markers for detecting LOH and/or amplification of chromosome segments. Bignell *et al*.[21] and Huang *et al*.[22] used Affymetrix SNPs genotyping arrays for determining the allelic state of the markers and for detecting DNA copy number changes. Compared with CGH, Affymetrix SNP arrays can be used to detect not only copy number changes but also genetic alterations with no copy number changes such as those caused by mitotic recombination. However, since most cancer cell lines do not have paired normal tissue, the question regarding what regions can be considered as regions affected by either LOH or amplification remain unsolved.

Recently we developed a novel high-throughput SNPs genotyping system.[23–25] With this system, users can customize their own SNP panels, more than 1,000 SNP-containing sequences can be amplified to a detectable amount in a single tube from as few as a single cell. Thousands of the amplified sequences containing SNPs can be resolved by a single microarray followed by genotype determination with high sensitivity, accuracy and simplified procedures. In the present study, this genotyping approach was used for detailed genetic analysis of 11 whole chromosomes and regions on two additional chromosomes in the MCF-7 breast cancer cell line using our customized SNP panel. By analyzing the results with our newly developed statistic approach, a number of chromosomal regions that may have been affected by LOH were revealed and localized to specific regions.

## SUBJECTS AND METHODS

### MCF-7 cell line and DNA samples

The MCF-7 cell line was kindly provided by Drs. William N. Hait/Jin-Ming Yang laboratory, which was originally from the laboratory of Dr. Kenneth Cowan of the Eppley Institute for Research in Cancer and Allied Diseases (Omaha, NE). The cell line was maintained in RPMI 1640 media containing 10% FBS, 100 units/ml penicillin, and 100 *μ*g/ml streptomycin at 37°C with 5% CO_2_ and 95% humidity. Genomic DNA was extracted with the TRIzol Reagents kit (Invitrogen, Inc.) according to the manufacturer's instructions and quantified by spectrophotometry. DNA of a control cell line, NB00637, which originated from a female Caucasian, and DNAs from 24 unrelated individuals from four ethnic groups, African American, Caucasian, Chinese and American Indian, six each, were purchased from the Coriell Cell Repositories (Camden, NJ)

### SNP selection

SNPs were selected from the dbSNP database (http://www.ncbi.nlm.nih.gov/SNP/index.html) maintained by the National Center for Biotechnology Information (NCBI). To use a two-color fluorescence (Cy3 and Cy5) labeling system for genotype determination, only transition SNPs (A/T to G/C changes or vice versa) were selected. A panel of 4,396 SNPs spanning 11 entire chromosomes and two regions on other chromosomes [Table 1] were incorporated into five multiplex groups, each of which contained 627 to 1172 SNPs. The majority of these SNP were described in our previous publications.[23,24]

**Table 1:** Comparison of SNP heterozygosities between the control cell lines and six normal individuals

Chromosome		1	2	6	13	14	15	16	17	18	20	21	22	X	Sum/Average
Total SNPs		666	516	807	207	215	196	343	318	365	109	545	60	49	4396
Covered region (Mb)		243.7	242.5	170.0	94.4	86.5	81.3	89.6	21.4	75.8	63.2	2.5	34.9	1.8	1207.5
SNPs/Mb		2.7	2.1	4.7	2.2	2.5	2.4	3.8	14.9	4.8	1.7	218.1	1.7	26.7	-
MCF7 cell line	A1*	313	219	328	101	88	99	159	157	187	45	255	29	23	2003
	A2*	250	181	282	91	66	64	116	109	172	46	200	12	24	1613
	A1+A2	65	106	177	0	51	31	49	33	2	17	9	17	0	557
	HR**	0.10	0.21	0.22	0.00	0.25	0.16	0.15	0.11	0.01	0.16	0.02	0.29	0.00	0.13
Control cell line	A1	248	203	316	87	86	96	142	133	143	47	195	28	20	1744
	A2	192	163	261	61	77	64	95	82	130	35	169	12	17	1358
	A1+A2	150	134	216	45	42	32	85	72	89	24	128	13	12	1042
	HR**	0.25	0.27	0.27	0.23	0.20	0.17	0.26	0.25	0.25	0.23	0.26	0.25	0.24	0.25
Six Caucasians	A1*	238	199	328	82	78	77	79	131	138	-	-	-	-	-
	A2*	160	155	233	64	62	59	55	85	113	-	-	-	-	-
	A1+A2	140	148	221	48	51	50	47	78	106	-	-	-	-	-
	HR*	0.260	0.295	0.282	0.246	0.268	0.269	0.261	0.266	0.296	-	-	-	-	-
*P* value***	MCF7 vs Ctrl	5.E-12	0.029	0.029	3.E-15	0.289	0.855	4.E-04	9.E-06	9.E-27	0.200	2.E-26	0.571	2.E-04	2.E-42
	Cauc. vs MCF7	3.E-12	0.002	0.009	3.E-16	0.660	0.009	0.003	1.E-06	1.E-33					
	Cauc. vs Ctrl	0.828	0.342	0.654	0.765	0.138	0.016	0.935	0.685	0.127	-	-	-	-	-

* A1 and A2, two SNP alleles.

** HR, heterozygosity rate.

*** χ^2^ Test was performed for most of group pairs. For those containing numbers less than 5, Fisher's Exact Test was performed, and two-tail results are shown.

### Primer and probe design

Primers and probes were designed using the software developed in our laboratory.[23] For each SNP, a pair of PCR primers was designed to amplify the sequence containing the polymorphism site. Another pair of primer-probes (so named because they can be used as either primers for generating single stranded DNAs (ssDNA) or probes printed on a slide for detecting the polymorphic sequences) was also designed. Primer-probes in each pair had their 3' ends immediately next to the same polymorphic site in the two different DNA strands. Since the primer-probes are internal (nested) with respected to the primers used for PCR products, they can be used to enhance the efficiency and specificity when used as primers. Twelve sets of oligonucleotides were used as positive probes and templates for quality control of hybridization and labeling. Each set consisting of three oligonucleotides, one was used as a probe and the other two as allelic templates differing by a single base. The sequences of those oligonucleotides were generated randomly by a computer program and were checked against the NCBI database to be certain that they didn't share significant sequence identity with any known human sequence in the database.

### Microarray preparation

Glass slides used for microarray were prepared according to the procedure described previously.[23] Briefly, pre-cleaned Gold Seal slides (Becton Dickson) were soaked in 30% bleach with shaking for 1 hour followed by rinsing six times with distilled water. The slides were then sonicated in 15% Fisher brand Versa-Clean Liquid Concentrate with heat on for 1 hour followed by rinsing 10 times with distilled water and five times with MilliQ water. Slides were dried by spinning in a microfuge at 1,000 rpm for 1-2 min, and then baked at 140°C in a vacuum oven (Fisher Scientific, Model 280A) for 4-6 hours. Before printing, 1 vol of each oligonucleotide probe solution were mixed with 4 vol of microarray printing solution to a final concentration of 40 *μ*M in a well of a 384-well plate. Probes were then spotted onto the washed glass slides by an OminGrid Accent microarray spotter (GeneMachines) under a humidity within a range of 50% to 55% at a temperature within a range of 22°C to 25°C. Each array consisted of 4 × 4 subarrays with 20 × 16 spots in each subarray. The total number of spots on the array was 5,056 including 4,602 SNP probes [Table 1], 246 negative controls (printing solution) and 208 positive controls.

### Multiplex PCR and ssDNA preparation

PCR was performed in a 30-*μ*l solution containing 1× PCR buffer (50 mM KCl, 100 mM Tris-HCl, pH 8.3, 1.5 mM MgCl_2_, and 100 *μ*g/ml gelatin), 250 *μ*M dNTPs (Invitrogen), 627-1172 pairs of primers (20 nM of each) for each multiplex group, 7.5 units of HotStar Taq DNA polymerase (Qiagen Inc.) and 50ng DNA. PCR samples were first heated to 94°C for 15 min to activate the Taq DNA polymerase followed by 40 PCR cycles. Each PCR cycle consisted of 40 sec at 94°C for denaturation, 2 min at 55°C followed by 5 min of ramping from 55°C to 70°C and 10 sec at 70°C for annealing and extension. A final extension was carried out at 72°C for 3 min after the cycles. PCR amplifications were performed in a T3 Thermocycler (Biometra). ssDNA was generated with 1.5 *μ*l of the multiplex PCR product from each group, one primer-probe for each SNP in 30 *μ*l PCR solution as described above, and 45 thermal cycles consisting of 30 sec at 94°C for denaturation, and 1.5 min at 55°C followed by 3 min of ramping from 55°C to 70°C and 10 sec at 70°C for annealing and extension.

### Hybridization

Eighty-five *μ*l of hybridization mixture containing 50 *μ*l of ssDNA (10 *μ*l from each group) and 35 *μ*l of hybridization solution (4.15 nM each positive control template oligo, 5× Denhart's solution, 0.5% SDS, and 4× SSC final concentrations) was added to cover a microarray under a cover slip. The slide was sealed in a Hybridization Chamber (Corning, NY), and the chamber was then soaked in a 56°C water bath for three hours. The slide was then taken out and washed at 56°C with 1× SSC and 0.1% SDS for 10 min, twice with 0.5× SSC for 30 sec and twice with 0.2× SSC for 30 sec.

### Probe labeling and microarray detection

The slide was spun to dry as above. The array was covered by 80 *μ*l of labeling solution containing 10.2 *μ*l of Sequenase buffer (supplied by the Sequenase vendor), 32 units of Sequenase (GE Healthcare, NJ), and 750 nM of Cy3-ddUTP and Cy5-ddCTP under a cover slip. Then, the slide was sealed in the chamber, immediately soaked in a 70°C water bath for 10 min, and then was washed again under the conditions used after hybridization as described above. After drying, the microarray slide was scanned by a GenePix 4000B microarray scanner (Axon Instruments) at 10 micron per pixel, 100% power and 480 to 560 PMT gain.

### Data analysis and genotyping

The microarray images from scanning were digitized with the computer program, GenePix Pro (Axon Instruments). Genotype calls for each SNP were determined from signal intensity data by a computer program developed in our laboratory as described previously.[26] After normalization and background subtraction, genotype calls were made by using the ratio between the two color intensities (Cy5/Cy3) with 0.4 and 2.5 as cutoffs. SNPs with ratios between or equal to the two cutoffs were assigned as heterozygous state and those with ratios outside of the range were assigned to the two respective homozygous states

## RESULTS

### Genotypes of the cell line MCF-7 and HB00637

**Figure 1:**
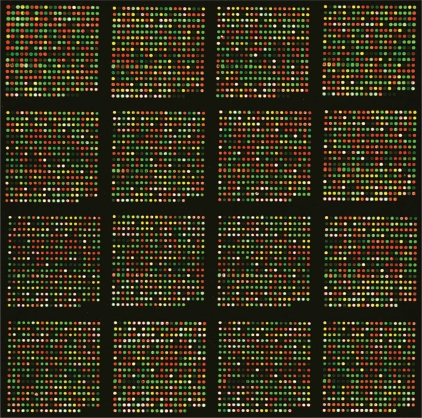
A two-color SNP microarray image containing 5,056 features from genotyping the MCF-7 cell line. Some of the 4,396 probes were printed twice. Spots that incorporated the Cy5-labeled ddCTP are in red (homozygous for the C allele); those that incorporated the Cy3-labeled ddUTP are in green (homozygous for the T allele); and the yellow spots indicate incorporation of both colors (heterozygous). White spots are those with over-saturating intensities but would not affect genotype determination.

Out of the 4,396 SNPs included in the study, 4,172 (94.9%) could be detected from the MCF-7 cell line [Figure 1] and 4,144 (94.3%) from the control cell line, NB00637. For the MCF-7 cell line 4,156 (94.5%) SNPs could be typed repeatedly with a concordant rate of 99.08%. Thirty-six SNPs were included at the time when SNPs were selected, but were not included in the later analysis because no information about their chromosomal positions is available in the current databases. As shown in Table 1, only 13.3% of SNPs were heterozygous in the MCF-7 cell line, whereas 25.1% in the control cell line. Such a significant difference (χ^2^ = 185.66, *P*<0.001) reflects a substantial genetic difference between the two cell lines.

Results from the MCF-7 cell line were also compared with those obtained from typing 24 human individuals from four ethnic groups (African American, Caucasian, Chinese and American Indian) analyzed with SNPs from Group I ∼ III covering nine chromosomes (1, 2, 6, 13-18). The average fraction of heterozygous SNPs was 26.3% for the four ethnic groups and 27.6% for Caucasian group alone, to which the donors of the two cell lines belong. The difference in fractions of heterozygous SNPs in the three multiplex groups between MCF-7 and the Caucasian panel analyzed was also significant (χ^2^ = 188.74, *P*<0.001).

Expected Size of Homozygous Zones in the Human Population. To further explore the cause of the differences between the fractions of heterozygous SNPs in different cell lines and in the human individuals, we examined the allelic states of the SNPs on individual chromosomes. Chromosomes with the fraction of heterozygous SNPs in MCF-7 significantly smaller than those in the control cell line and in the Caucasian individuals could be affected by LOH or amplification. As shown in Table 1, no chromosome except for chromosome 15 in the control cell line had a fraction of heterozygous SNPs that was significantly different from the Caucasian individuals. The fraction of heterozygous SNPs on chromosome 15 in the control cell line was significantly smaller than that in the Caucasian individuals reflects certain abnormality in this cell line. Differences between the fractions of heterozygous SNPs for chromosomes 14, 15, 20 and 22 of the two cell lines were not significant (*P*>0.05, χ^2^-test).

For most cancer cell lines, it is difficult or impossible to obtain the paired normal tissue from the donors. To identify chromosomal regions possibly affected by loss of heterozygosity, we developed an effective method based on both statistic and genetic considerations. First, we assume that there is no linkage disequilibrium (LD) between the SNPs included in the present study. Genetically, when markers are in LD that is often the case when markers are located closely, certain alleles of the SNPs are associated from generation to generation in the population. Since SNPs used in the present study were 220 to 500 KB apart, and LD is usually not observed between markers separated by such distances, the alleles of the SNPs selected in the present study should be random combined in the human population. When the frequencies of the alleles of a group of SNPs in a given human population is known, the fractions of the homozygous and heterozygous individuals with respect to these SNPs can be estimated in this population. Statistically, the maximal number of the clustering homozygous SNPs is a function of the fraction of homozygous SNPs in the population and the number of SNPs in the regions under consideration, and can be calculated based on binomial distribution. Although the mathematical approach for the calculation was described by Philippou and Muwafi in 1982,[27] since the procedure involves complicated and an excessive amount of computation. To make the computation practically feasible, we developed a very straight forward simulation approach for analyzing the data from the present study.

Since the MCF-7 donor was a Caucasian, the average fraction of homozygous SNPs, which is 72.4% in the Caucasian population, was used for the analysis. Because only SNPs on the same chromosome may form homozygous SNP clusters or Homozygous Zones (HZs), the maximal size of HZs that may be found based on random combination was calculated for each chromosome separately based on the number of SNPs used in the present study. As defined by the simulation method, the maximal number homozygous SNPs in each cluster from random combination is calculated based on a probability of 0.01. Results are listed in Table 2 (see the column “Max Number of SNPs in a Random HZ”).

**Table 2:** Homozygous zone with possible loss of heterozygosity in the cell line MCF-7

Chromosome	No. of SNPs	Exp'ed max No. of SNPs in an HZ*	Homozygous zone					
			
			Start SNP		End SNP		No. of SNPs	Size (Mb)
						
			RS#	Position (bp)	RS#	Position (bp)		
1	666	30	912842	19105102	1570685	33582439	43	14.477
			1478928	34185838	1802762	47361584	35	13.176
			1342686	72748861	1574509	109133972	100	36.385
			2030027	215538831	1782212	245037793	80	29.499
2	516	30	-	-	-	-	-	--
6	807	31	284416	157368575	760501	170641806	68	13.273
13	207	26	995813	18449159	5960	112849738	192	94.401
14	215	26	-	-	-	-	-	--
15	196	26	2115636	19837058	907437	52425732	68	32.589
16	343	28	2229654	29582827	899227	54944970	51	25.362
			154439	57244101	2230095	66714431	41	9.470
17	318	28	315440	27063949	966957	49353946	74	22.290
			759495	69511996	1471196	80481532	43	10.970
18	365	28	507184	176998	570514	20365062	94	20.188
			193156	20581 1 36	2469895	30366403	43	9.785
			595309	30816400	575682	75945510	222	45.129
20	109	24	-	-	-	-	-	-
21	545	30	283591 3	38018385	986363	38451807	77	0.433
			2836175	38460419	2836462	38817670	69	0.357
			1888468	38886616	1524930	39890330	167	1.004
22	60	22	-	-	-	-	-	-
X	49	21	2692989	31184565	982767	33887741	47	2.703
Total/Average	4396						83.37	20.097

* Since the numbers of SNPs are whole numbers, the numbers in this column are the integers closest to the exact values calculated using *p*=0.01. HZ, homozygous zone.

Fourteen HZs were found with size greater than the maximum number of homozygous SNPs expected based on random combination on nine chromosomes, 1, 2, 6, 13-18, 20 and X in the MCF-7 cell line [Table 2] and none were found in the HB00637 control cell line. The number of SNPs in these HZs ranged from 30 to 359. No heterozygous SNPs were found along the entire length of chromosome 13. Only two heterozygous SNPs were found on the entire chromosome 18. One HZ on chromosome 16 was found across the centromere.

**Figure 2:**
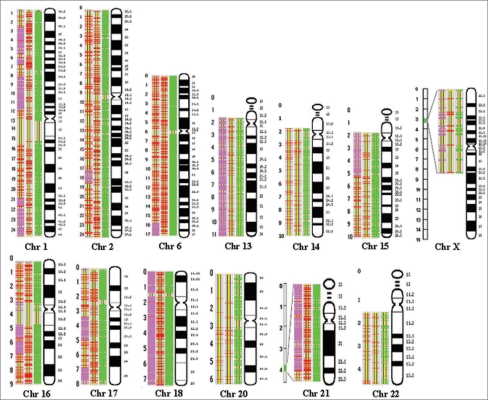
Detected homozygous zones. Columns for each chromosome from right to left: the chromosome, available SNPs on the chromosome, heterozygous SNPs in the control cell line, heterozygous SNPs and homozygous zones in MCF-7. Horizontal slashes in each column: green, available SNPs; red, heterozygous SNPs; orchid, homozygous SNPs in HZs. Numbers on the right side, cytogenetic band numbers, and those on the left side, distance/10,000,000 (in bp) from the telomere of the short arm on chromosome.

The analyzed regions on chromosomes 21 and X were small (2.5 and 1.8 Mb, respectively) with very high SNP densities (218 and 26.7 SNPs/Mb, respectively). Since closely located markers are often found in LD, the assumption that markers are randomly associated may not be applied to these two regions. Therefore, the HZ in these regions may not be defined by the criteria described above. However, the entire examined region with 47 homozygous SNPs spun 1.8 Mb on chromosome X and a region with 397 affected SNPs covered a major portion (2.24 Mb) of the 2.5-Mb region on chromosome 21. Based on the data reported, the size of the haplotype blocks (genomic regions with markers in LD) in the human genome is estimated to be ∼8 kb.[28–32] The 1.8 Mb region on chromosome X hereby may contain ∼225 haplotype blocks and the 2.24 Mb region on chromosome 21 may have >279 haplotype blocks. The probability of having so many homozygous SNPs in >200 haplotype blocks would be very low. To further explore the properties of the homozygous zones in these small regions, we compared the result from chromosome 21 in MCF-7 with those obtained from 16 Caucasian samples analyzed with the same multiplex group in another study (Greenawalt *et al*, unpublished data). No HZ of comparable size was found from the 16 samples. Therefore, the HZ with 397 SNPs and 2.24 Mb on chromosome 21 in MCF-7 should be considered as nonrandom. Since the HZ identified on chromosome 21 extends to the end of the 2.5-Mb region, it is likely that the entire HZ could be larger than what we can learn from the present study. Four SNPs in this zone were detected as heterozygous. However, we have shown that a significant portion (up to 4.4%) of the SNPs in the version of database used for SNP selection may not be real polymorphism site and may be paralogous sequence variants (Luo *et al*, unpublished data), the percentage (4/430 = 0.93%) of heterozygous SNPs in this HZ are within this range. The positions and distribution of all HZs in MCF-7 are shown in Figure 2.

## DISCUSSION

In most cancer genetic studies, genetic alterations in the tumor cells can be identified by comparing the genotypes of tumor cells with those for paired normal tissue. However, it is difficult or impossible to obtain paired normal tissue for many of the cancer cell lines, especially for those that were established a long time ago. Negrini *et al*[33] reported that five polymorphic markers (microsatellite) on chromosome 11 were homozygous in MCF-7 cell line and the calculated probability of these five markers all being homozygous was less than 1%. It was concluded that one copy of chromosome 11, or at least the short arm of it, in MCF-7 was lost. In the present study, we used our recently developed statistical approach and much higher marker density, and identified the HZs that may not be caused by random combination in the breast cancer cell line, MCF-7. Fourteen HZs were found in the cell line but none in the control cell line. Another two HZs covered the entire or nearly entire examined regions on chromosome X and 21 in MCF-7 with a high marker density for which the expectation based on random combination of SNP alleles also may not apply.

It should be pointed out that HZs larger than expected may not be necessarily caused by LOH or chromosomal segment amplification. The presence of HZs with sizes significantly greater than expected random combination in the human population were first reported by Broman and Weber.[34] The authors analyzed 134 individuals from eight CEPH (the Centre d'E´ tude du Polymorphisme Humain) families. All individuals in two (25%) of the families were shown to have at least one segment of homozygosity, and 20% of the individuals in the other six families also had significant homozygous segments. Therefore, when HZs are identified in cancer cells, one cannot simply conclude that these are all caused by genetic alterations in somatic cells unless the genotypes of paired normal tissues are available for comparison. However, the majority of HZs identified in the present study should be different from those identified in the study by Broman and Weber since their study indicates that the fractions of families and individuals among the samples analyzed may be present only in a small portion of human individuals. Families with HZs in all of their members may be caused by particular reasons such as marriage of close relatives. If the HZs in the two families were caused by marriage of close relatives, the sizes of these HZs should be larger than those present in the general population. However, the average size of the HZs in the two families were 10.9 and 18.5 Mb many of which were less than 5 Mb, compared to the averages size of 26.9 Mb with a minimal size of 9.5 Mb identified in the present study.

More intensive analysis using results from the Human HapMap Project was reported very recently.[35] Their results should be more accurate and more representative of the human population than those described in Broman and Weber because (1) all markers used are SNPs that are more stable than microsatellite markers used in Broman and Weber; (2) 209 HapMap individuals, the majority of which were unrelated were used, instead of 134 individuals in eight families used in Broman and Weber; and (3) much high marker density (one SNP every 500 bp). The authors identified 1393 tracts exceeding 1 Mb in length. However, HZs identified in that study were even more different from those identified in the presents study in two aspects: (1) HZs were defined as uninterrupted clusters of homozygous SNPs spanning at least 1 Mb in a single individual, which allowed authors to study HZs with different criterion but has less statistical consideration compared with the present study; and (2) among the 1,393 tracts identified, only 17 were longer than 5 Mb. The longest HZ was only 17.9 Mb, which is shorter than eight of the 14 HZs identified in the present study [Table 2].

The presence of significantly more HZs in the MCF-7 cell line than in the human population indicates that it is very likely that the majority of these HZs are the results of LOH. Our results are further validated by comparing with those reported by others using other methods. Most of the regions of DNA loss or chromosomal deletion identified in the literatures were similar to what we identified in the present study. For example, LOHs of 1p (19.11 - 33.58 Mb, 34.19 - 47.36 Mb, and 72.75 - 109.13 Mb), 1q (215.54 - 245.04 Mb), 6q (157.37 – 170.64 Mb), 13 (18.45 - 112.85 Mb), 15q (19.84 - 52.43 Mb), 16q (29.58 - 54.94 Mb and 57.24 – 66.71 Mb), 18 (nearly whole chromosome loss), and 21q (38.02 – 40.52 Mb) were reported by several studies using CGH[4,10,14,15] and karyotyping.[2,3] These results indicate that our method for detecting HZs in cell lines is very reliable.

Comparing the results from CGH studies on MCF-7 reported by others, several HZs found from our study are of particular interest. Two HZs on 17q (27.06 - 49.35 Mb and 69.51 - 80.48 Mb) detected in our study were shown to be amplified by others using CGH[15]. Although the HZ from 171.40 to 184.09 Mb on 2q is smaller than the predicted minimum size (26 homologous SNPs in cluster compared to 30 for the minimum size of a non-random zone) it was shown to be amplified in some sub-lines of MCF-7.[9,13–15,36] These findings provide evidence for the hypothesis that HZs may be detected not only in the chromosomal regions affected by loss of one of the homologue copies and in regions showing non-randomness underlain by other genetic alterations but also in those affected by amplification of one allele or disproportionate amplification of both alleles.

Discrepancies were found between our results and those reported previously. HZs on 17p in the MCF-7 cell line was reported by other studies[14,15] using CGH but was not detected in our study. In addition, we did not detect any alteration on chromosomes 20 and 22, while amplification of 20q and loss of 22q were observed by others.[14,15] One of the possible reasons for these discrepancies could be that the cell line MCF-7 used in different studies may have diverged genetically. Several studies[4,14,15] reported that there was considerable genetic variation among different cultures of the MCF-7 cell line or MCF-7 from different sources. This brings up an important issue that is how to interpret and compare experimental data collected from other biological studies such as drug resistance, gene expression profiling and pathway analysis by different laboratories using the MCF-7 cell line or any other lines from different sources. If a cell line has been cultured by different laboratories for a long period of time, different genetic alterations may have arisen. Results from studies with these cultures could be different. For amplification on chromosome 20q detected by others but not by us, the discrepancy could be because the amplified copy numbers of the two alleles were equal or nearly equal so that our analysis was not able to detect such difference. Indeed, it is interesting to learn that when a chromosomal region is amplified, whether both alleles would be equally, unequally or selectively amplified.

Unlike CGH that can be used to determine DNA copy number (loss or amplification) changes but can't discriminate copy number changes of different alleles, genotype analysis can be used to detect both LOH[37,38] and amplification of individual alleles[21,39]. Genotype analysis can also be used to detect LOH that may not necessarily involve DNA copy number changes,[21] which cannot be detected by CGH. With our method polymorphic sequences can be amplified and used for analyzing samples from very small quantity or even very few copies,[23] while sophisticated whole genome amplification without bias is required for CGH when a small amount of material is used. Resolution was a major issue for CGH, which has been improved by development of microarray based CGH. With genotype analysis, very high resolution can be reached as long as markers are available. Therefore, genetic analysis and CGH may be used as complementary approaches for large-scale genetic profiling

## CONCLUSIONS

In summary, since many cell lines such as MCF-7 are widely used in biomedical research, learning the genetic backgrounds of these lines and the genetic variation within each cell line from different sources is very important to understand experimental results on a genetic basis and for interpret experimental data correctly. Our system allows genetic profiling of widely used cell lines with a high marker density on a periodical basis to minor genetic changes occurring during passages and/or maintenance in different laboratories.
